# The Function of *Drosophila* USP14 in Endoplasmic Reticulum Stress and Retinal Degeneration in a Model for Autosomal Dominant Retinitis Pigmentosa

**DOI:** 10.3390/biology9100332

**Published:** 2020-10-12

**Authors:** Jung-Eun Park, Thị Xuân Thùy Trần, Nayoung Park, Jeonghun Yeom, Kyunggon Kim, Min-Ji Kang

**Affiliations:** 1 Department of Biomedical Sciences, Asan Medical Center, University of Ulsan College of Medicine, Seoul 05505, Korea; pje870519@gmail.com (J.-E.P.); ttxthuy.1994@gmail.com (T.X.T.T.); skqhdqhd1234@gmail.com (N.P.); 2 Convergence Medicine Research Center, Asan Institute for Life Sciences, Asan Medical Center, 88-gil, 43 Olympic-ro, Songpa-gu, Seoul 05505, Korea; nature8309@gmail.com (J.Y.); kimkyunggon@gmail.com (K.K.)

**Keywords:** USP14, ER stress, retinal degeneration, *Drosophila*

## Abstract

**Simple Summary:**

The present study shows the role of *Drosophila* USP14 under ER stress and ER stress related disease, autosomal dominant retinitis pigmentosa. *Drosophila* USP14 protects cell from ER stress triggered by ER stress-causing chemicals *Drosophila* S2 cells and suppresses the retinal degeneration in disease model for retinitis pigmentosa by regulating the stability of Rhodopsin-1. This study also indicates the dynamic reorganization of proteasome complex under ER stress. The modulation of USP14 could be a potential therapeutic strategy for treating the diseases associated with protein folding.

**Abstract:**

Endoplasmic reticulum (ER) stress and its adaptive cellular response, the unfolded protein response (UPR), are involved in various diseases including neurodegenerative diseases, metabolic diseases, and even cancers. Here, we analyzed the novel function of ubiquitin-specific peptidase 14 (USP14) in ER stress. The overexpression of *Drosophila* USP14 protected the cells from ER stress without affecting the proteasomal activity. Null Hong Kong (NHK) and alpha-1-antitrypsin Z (ATZ) are ER-associated degradation substrates. The degradation of NHK, but not of ATZ, was delayed by USP14. USP14 restored the levels of rhodopsin-1 protein in a *Drosophila* model for autosomal dominant retinitis pigmentosa and suppressed the retinal degeneration in this model. In addition, we observed that proteasome complex is dynamically reorganized in response to ER stress in human 293T cells. These findings suggest that USP14 may be a therapeutic strategy in diseases associated with ER stress.

## 1. Introduction

The endoplasmic reticulum (ER) is a major organelle in which membrane and secretory proteins are synthesized and folded properly. When the rate of protein synthesis exceeds the folding capacity of the ER under certain conditions, misfolded or unfolded proteins accumulate in the ER [1]. To overcome this stress, cells activate a signaling pathway referred to as unfolded protein response (UPR). The UPR pathway is executed by three ER membrane-embedded sensors: inositol-requiring enzyme 1 (IRE1), activating transcription factor 6 (ATF6), and double-stranded RNA-dependent protein kinase (PKR)-like ER kinase (PERK). These three sensors trigger transcriptional activation mediated by three distinct transducers, spliced X-Box Binding Protein 1 (sXBP1), cleaved ATF6, and ATF4, respectively. These proteins trigger the transcription of genes including those encoding chaperones and ER-associated degradation (ERAD) pathway to restore ER function [2,3,4]. However, when ER homeostasis cannot be restored, the cell death program is activated, resulting in the development of various diseases, including neurodegenerative disease, retinitis pigmentosa, and diabetes. 

The ubiquitin-proteasome system (UPS) is the principle proteolytic protein quality control system. It mainly regulates the short-lived proteins levels in cells [5,6]. The target substrate for the UPS is covalently conjugated to ubiquitin via an enzymatic reaction that involves three distinct proteins: E1s, E2s, and E3s. Ubiquitinated proteins bind to the 26S proteasome complex for degradation. While the ATP-dependent core 20S proteasome degrades the polypeptide, one or two 19S proteasome regulatory particles bind to the ubiquitinated substrate. Deubiquitinating enzymes (DUBs) associated with 19S, such as USP14, UCHL5/UCH37, and RPN11, remove the ubiquitin moieties conjugated to the substrate prior to the translocation of the substrate to the catalytic chamber of the 20S proteasome [7]. UPS is closely associated with the removal of misfolded or unfolded proteins accumulated in the ER by the ERAD. Hrd1 is an E3 ubiquitin ligase that constitutes ERAD and forms a complex with Hrd3 and Derlin1 to provide a channel for the retrotranslocation of misfolded proteins from the ER to the cytosol, where misfolded proteins are degraded by proteasomes. 

In this study, we report the function of *Drosophila* USP14 in ER stress. Overexpression of USP14 reduced the level of ER stress, estimated by the induction of the UPR markers hsc3 and ATF4 in *Drosophila* S2 cells, whereas the knockdown of USP14 using dsRNA increased the levels of ER stress markers. Consistently, the activation of ER stress reporters, namely, *xbp1_p_* > *dsRed* and *4E-BP^intron^* > *dsRed*, by tunicamycin (TM) was reduced upon the overexpression of USP14. *Drosophila* USP14 utilized specific substrates for proteasomal degradation and regulated the stability of Null Hong Kong (NHK)—but not ATZ—variants harboring mutant alleles of alpha-1-antitrypsin. Overexpression of USP14 restored the levels of rhodopsin-1 (Rh-1) protein in the *Drosophila* model of autosomal dominant retinitis pigmentosa (ADRP) and suppressed the retinal degeneration in the model. We also analyzed the proteasomal dynamics during ER stress. These results suggest that the manipulation of USP14 can be developed as a therapeutic strategy in diseases associated with protein conformation.

## 2. Materials and Methods

### 2.1. Plasmids and Fly Strains

The coding sequence of USP14 was obtained by RT-PCR from *yw* larvae, and it was tagged with hemagglutinin (HA) at the C-terminus. HA-tagged USP14 was subcloned into pUAST vector [8]. All *Drosophila* stocks were maintained on standard BDSC cornmeal containing 1.6% yeast, 0.9% soy flour, 6.7% cornmeal, 1% agar, and 7% light corn syrup at 25 °C. Gal4/UAS system was used to express the target genes in *Drosophila*. Those fly lines were used for this study: *actin-Gal4* [9], *UAS-NHK* [10], *UAS-ATZ* [10], *GMR-Gal4* [11], *Rh1-Gal4*, *Rh1-GFP* [12], *ninaE^G69D^/TM6B* [13], and *UAS-dicer2* [14].

### 2.2. Cell Culture and dsRNA Treatment

*Drosophila* S2 cells were cultured in Schneider’s *Drosophila* medium (Invitrogen, 21720, Waltham, MA, USA) supplemented with 10% fetal bovine serum (Invitrogen, 16000-044, Waltham, MA, USA) and 0.5% penicillin/streptomycin (Invitrogen, 15140-122, Waltham, MA, USA). The double-stranded RNA (dsRNA) treatment was carried out by standard protocol [15].

### 2.3. Western Blotting

*Drosophila* S2 cells were lysed using 1% sodium dodecyl sulfate (SDS) buffer containing 10 mM Tris pH 7.5, 150 mM NaCl, 1 mM EDTA, 1% SDS, and protease inhibitor (Roche). After centrifugation at 15,700 g for 10 min, the proteins in the supernatant were separated by SDS-PAGE and transferred onto a polyvinylidene difluoride membrane (Merck Millipore, Billerica, MA, USA). The antibodies used in this study are listed in Supplementary Table S3.

### 2.4. RT-PCR and Real-Time RT-PCR

Total RNA was isolated using TRIzol (Invitrogen, Waltham, MA, USA), and 100 ng of RNA was transcribed with ReverTra Ace qPCR RT kit. PCR was done by the following program using taq polymerase (Roche) on C1000 Touch^TM^ Thermal Cycler (Bio-Rad): 94 °C for 5′, 94 °C for 30″, 57 °C for 30″, 72 °C for 30″ (total 30 cycles), and 72 °C for 5′. The real-time PCR was run for 40 cycles using the TOPreal^TM^ qPCR 2X PreMIX (SYBR Green with high ROX) and a LightCycler 480 Real-Time PCR system (Roche). The following primer sequences were used: A1AT_F 5′- GGCTGACACTCACGATGAAA, A1AT_R 5′- GTGTCCCCGAAGTTGACAGT, ninaE_F 5′- TCATCATTGCTGCTGTCTCC, ninaE_R 5′- CCCCAAATGGTATTCAGTGG, -tubulin_F 5′- CGAGACCTACTGCATCGACA, -tubulin_R 5′- AGGTCACCGTATGTGGGTGT, usp14_F 5′- CGTGCTACATGAATGCCACT, usp14-F 5′- TCGACATAGTGTCGGTTCCA, rp49_F 5′- AGATCGTGAAGAAGGCACCAAG, rp49-R 5′- CACCAGGAACTTCTTGAATCCGG.

### 2.5. Measurement of Proteasomal Activity

The fluorogenic proteasome substrate N-succinyl-Leu-Leu-Val-Tyr-7-aminomethylcoumarin (suc-LLVY-AMC, Enzo Life Sciences, USA; BML-P802) was used to determine proteasome activity. The hydrolysis of substrates was measured with 15 μg of whole-cell extracts (WCEs) and 50 μM of suc-LLVY-AMC. The reaction was done in the assay buffer (50 mM Tris pH 7.5; 1 mM EDTA; 1 mg/ml bovine serum albumin; 1 mM ATP; 1 mM dithiothreitol). Fluorescence was measured for 4 h with excitation and emission wavelengths of 380 nm and 460 nm, respectively.

### 2.6. Analysis of Retinal Degeneration

As an eye pigments may affect the retinal degeneration, all flies expressed an RNA interference (RNAi) against the white gene (Bloomington Drosophila Stock Center #33613, Bloomington, Indiana, IN, USA). The selected flies were reared in vials (20‒30 flies in each vial) under permanent light at 25 °C. The vials were changed every 2 days. Pseudopupils were quantified on a pad under blue fluorescent light after anesthetizing the flies with CO_2_.

### 2.7. Purification of 26S Proteasomes

The purification of Human proteasomes was performed from HEK293 cells expressing biotin-tagged human 4 subunit, as previously described [16]. The HEK293 cells were treated with 5 μg/ml of tunicamycin for 6 h. For pre-treatment experiments, the HEK293 cells were incubated for 10 h with 50 μM IU1, followed by treatment with 5 μg/ml of tunicamycin for 6 h. The cells were lysed in the buffer (50 mM NaH_2_PO_4_ pH 7.5; 5 mM MgCl_2_; 100 mM NaCl; 10% glycerol; 0.5% NP-40; 5 mM ATP; 1 mM DTT) containing protease inhibitor and homogenized with a Dounce homogenizer. The proteasome complex was isolated using agarose beads (Millipore, Billerica, MA, USA). The beads were then washed four times with lysis buffer and TEV buffer (50 mM Tris-HCl pH 7.5; 1 mM ATP; 10% glycerol). The proteasomes were eluted using TEV protease (Thermo Fisher, Waltham, MA, USA). The elutes were concentrated using Amicon ultra centrifugal filters (Millipore, Billerica, MA, USA).

### 2.8. Sample Preparation for Proteomic Analysis

Samples from immunoprecipitation were firstly lyophilized in a centrifugal vacuum concentrator (LABCONCO, CentriVap, Kansas City, MO, USA) to reduce volume. Dried samples were reconstituted with 100 μL of 50 mM triethylammonium bicarbonate (pH 7.55, Thermo Fisher Scientific) with 5% sodium dodecyl sulfate. Proteins were digested by S trap-based enzymetic digestion method as described previously [17], except trypsin/LysC protease mixture (Promega) was used at the digestion step.

### 2.9. Mass Spectrometry Analysis with Liquid Chromatography

Peptide mixtures were separated using nano-LC system (UltiMate 3000 RSLC, Thermo Scientific, Waltham, MA, USA) and reversed-phase chromatography. Peptides samples were trapped on trap column (Thermo Scientific, USA) and peptide separation was conducted on C18 analytical column (Thermo Scientific, USA). Peptides were eluted using a 150-min gradient using buffer A (5% dimethyl sulfoxide with 0.1% formic acid) and buffer B (5% dimethyl sulfoxide with 0.1% formic acid in 80% acetonitrile) and [18] at 50 °C (buffer B from 5 to 40% over 150 min, from 40 to 95% over 2 min, and kept at 95% for 23 min, then from 95 to 5% over 10 min and kept at 5% for 15 min) at 250 nL/min. The mass spectrometry analysis was performed using Q Exactive Plus mass spectrometer (Thermo Scientific). Data-dependent acquisition was performed with an automatic switch between a MS1 (*m*/*z* 350-1800) and 20 MS2 scans. MS1 spectra were measured with an AGC of 3e6, a resolution of 70,000 and an injection time of 100 ms. MS/MS spectra were triggered at an AGC of 1e5, a resolution of 17,500, and a normalized collision energy of 27. Repeated peptides were excluded for 20 s. Three technical replicates were applied for each sample.

### 2.10. Database Searching and Label-Free Quantitation

All MS data were searched against the SwissProt sequence database (Jun 2019) using the SequestHT on Proteome Discoverer (version 2.2, Thermo Scientific). MS2 spectra were searched using a precursor ion mass tolerance of ± 10 ppm and MS/MS mass tolerance of 0.02 Da. False discovery rates (FDRs) were set for 1% for each analysis using “Percolator”. Other search parameters were set as default. Relative quantitation (label-free) was performed using peak intensity for razor peptide and a unique peptide of each protein. Normalization was performed with total peptide amount of each sample set. An imputation was processed with a replicate-based resampling method, which imputes missed abundance values within each group with random abundance values picked around the median abundance of replicates.

### 2.11. Statistical Analysis

The free software Perseus (https://maxquant.net/perseus/, version 1.6.14.0, Max-Planck-Institute of Biochemistry, Germany) was applied to analyze the relative abundance of proteins among samples. The normalized protein abundance values were transformed to log_2_ values. Technical triplicates of each set were set as a group, and a minimum of three valid values was required in at least one group. Missing abundance values were changed by random values from a normal distribution with default parameters (downshift: 1.8, width: 0.3). To assess the quality of our datasets, Perseus software was used for principal component analysis (PCA). Student’s t-test was applied using Benjamini–Hochberg FDR with 0.05 cut-off to find statistically significant differentially expressed proteins (DEPs) between samples. A volcano plot was constructed using log_2_ value of ratio against -log10 of q-value and visualized using InstantClue software [19]. Biological processes (GO-terms) were performed for the functional categorization of differentially associated proteins using Enrichr [20]. We selected an adjusted *p*-value of ≤0.01 as the cutoff value. All other experiments were repeated at least three times, and resultant data were presented as means with standard errors of the means.

## 3. Results

### 3.1. Drosophila USP14 Regulates ER Stress in Drosophila S2 Cells.

**Figure 1:**
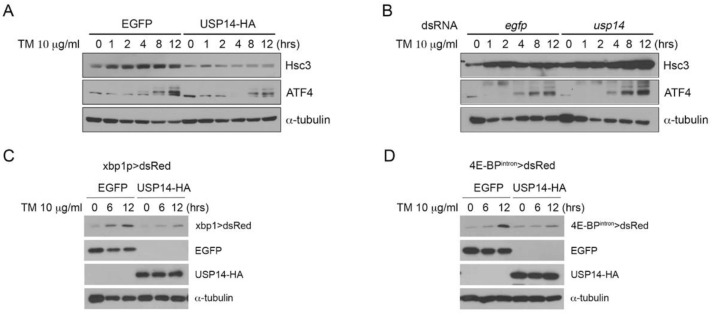
*Drosophila* USP14 has a protective role in endoplasmic reticulum (ER) stress. (**A**) Enhanced green fluorescent protein (EGFP, control) or USP14-HA was transiently transfected into *Drosophila* S2 cells. The transfected cells were treated with 10 μg/ml tunicamycin (TM) for the indicated intervals. The levels of the unfolded protein response (UPR) markers hsc3 and AFT4 were increased upon TM treatment and reduced upon USP14 overexpression. (**B**) The knockdown of USP14 by dsRNA increased the levels of hsc3 or ATF4 compared to those in the control cells (*egfp* dsRNA transfection). (**C**) *xbp1* > *dsRed* reporter expression was detected via Western blotting after the treatment with TM. The above numbers indicate the duration of TM treatment (hours). (**D**) *4E-BP^intron^ > dsRed* reporter expression upon TM treatment.

ER stress is closely associated with the ubiquitin-proteasome system that acts via ERAD. To determine whether USP14 regulates the levels of ER stress, we first overexpressed *Drosophila* USP14 with HA epitope tags in *Drosophila* S2 cells. The degree of ER stress in the cells was assessed based on the extent of the activation of UPR markers, such as hsc3 and ATF4. When S2 cells were treated with TM for 12 h, the expression of the ER chaperone hsc3, which is the *Drosophila* homolog of GRP78/BiP, was induced after 1 h (Figure 1A). However, overexpression of USP14 reduced the levels of hsc3 induction via ER stress (Figure 1A and Supplementary Figure S1A). We also assessed the activation PERK pathway using the anti-ATF4 antibody. Similar to hsc3, ATF4 induction by TM was suppressed in USP14-overexpressing cells (Figure 1A and Supplementary Figure S1A), indicating that USP14 is involved in ER stress. Similar results were obtained after treatment with 1 M thapsigargin, which induces ER stress by depleting the calcium content in the ER (Supplementary Figure S2). We also examined the levels of ER stress after USP14 knockdown. The levels of *usp14* in usp14 dsRNA-treated S2 cells decreased by approximately 50% compared to the control cells (Supplementary Figure S3). Interestingly, the basal level of hsc3 in USP14 knockdown cells was higher than that in control cells (Figure 1B and Supplementary Figure S1B). The induction of hsc3 expression by TM was increased in USP14 knockdown cells (Figure 1B and Supplementary Figure S1B). Likewise, TM-induced ATF4 levels were also increased upon USP14 knockdown (Figure 1B and Supplementary Figure S1B). Next, we estimated the levels of ER stress using a previously established ER-stress reporter in *Drosophila*, namely, *xbp1_p_ > dsRed*, which was designed to detect the transcriptional activation of xbp1 [21]. The activation of the ER stress reporter was assessed based on the expression of dsRed. *xbp1_p_ > dsRed* expression was increased by ER stress caused by TM treatment. However, the activation of this reporter was reduced upon the overexpression of USP14 (Figure 1C). The levels of ER stress were assessed using another ER stress reporter, *4E-BP^intron^ > dsRed*. This reporter contains ATF4-binding sites in 4E-BP intron sequences, which not only respond to ER stress but also to induction of ATF4 [22]. The induction of reporter by TM was highly reduced in USP14-overexpressing S2 cells (Figure 1D).

As *Drosophila* USP14 suppressed the level of TM-induced ER stress, we next tested whether the protective role of USP14 under conditions of ER stress can be attributed to the regulation of proteasomal activity by USP14. To assess the proteasomal activity, we used the proteasome substrate suc-LLVY-AMC in *Drosophila* S2 cells. Treatment of enhanced green fluorescent protein (EGFP)-overexpressing S2 cells with 10 μg/ml TM caused a significant reduction in the fluorescent signals corresponding to the overall peptidase activity of the 26S proteasome within 1 h (Supplementary Figure S4). The levels of the overall 26S proteasome activity in USP14-overexpressing cells were similar to those in the control cells (Supplementary Figure S4). Collectively, these results indicate that *Drosophila* USP14 protects cells from ER stress, but this process does not involve regulation of proteasomal activity.

### 3.2. Drosophila USP14 Regulates the Degradation of Mutant A1AT Variants

Proteasome aids the ER quality control by degrading misfolded proteins in the ER lumen. To investigate whether *Drosophila* USP14 degrades ERAD substrates, we examined the effect of USP14 on the degradation of the misfolded alpha 1-antitrypsin variants ATZ and NHK, which are well-known ERAD substrates. However, the two alleles of A1AT show different biochemical characteristics as NHK activates xbp1 mRNA splicing while ATZ variants do not activate UPR [10,23].

**Figure 2:**
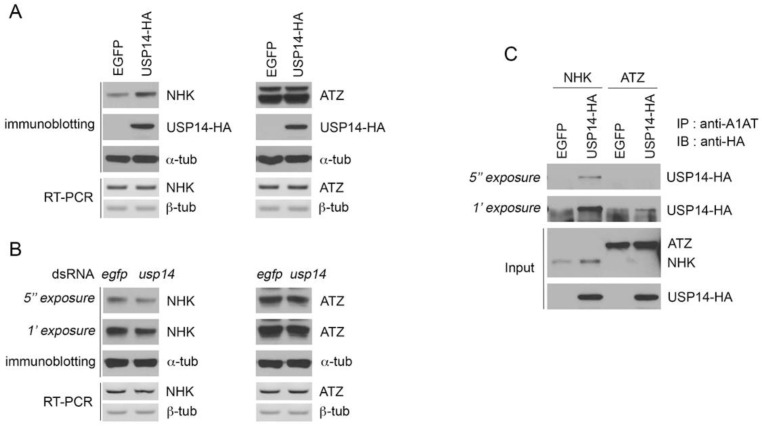
*Drosophila* USP14 regulates the stability of mutant A1AT proteins. (**A**) Levels of Null Hong Kong (NHK)—but not ATZ—were increased upon the overexpression of USP14. The A1AT variants were co-expressed with EGFP or USP14-HA and the protein levels of NHK or ATZ variants were determined by immunoblotting. The mRNA levels of NHK and ATZ were examined by RT-PCR. (**B**) NHK protein degradation was accelerated upon USP14 knockdown. NHK or ATZ was transfected in dsRNA against *egfp* (negative control) or *usp14* pre-treated *Drosophila* S2 cells. The levels of mutant A1AT were determined using the anti-A1AT antibody. (**C**) The interaction of NHK with USP14. *Drosophila* S2 cells were transfected with NHK or ATZ together with EGFP or hemagglutinin (HA)-tagged USP14. The protein complexes were purified using the anti-A1AT antibody and analyzed by immunoblotting using the anti-HA antibody. IP, immunoprecipitated; IB, immunoblot.

The expression of NHK increased in response to USP14 overexpression in *Drosophila* S2 cells (Figure 2A). However, the overexpression of *Drosophila* USP14 did not affect the expression of ATZ variants in *Drosophila* S2 cells. These findings indicate that *Drosophila* USP14 is not involved in the degradation of misfolded ATZ variants. In addition, we downregulated the expression of USP14 in *Drosophila* S2 cells using dsRNA against USP14; NHK degradation was accelerated upon USP14 knockdown. However, this knockdown did not affect the stability of the ATZ variants (Figure 2B). There were no changes in the mRNA levels of NHK or ATZ in USP14 overexpression or knockdown conditions (Figure 2A,B, RT-PCR). Subsequently, a co-immunoprecipitation assay was performed to test whether USP14 interacts with either the NHK or ATZ variant. As shown in Figure 2C, the interaction between NHK and USP14 was readily detected when NHK and HA-tagged USP14 were co-expressed in *Drosophila* S2 cells. Consistent with the weak effect of USP14 on the ATZ level, the interaction between ATZ and HA-tagged USP14 was negligible (Figure 2C). Taken together, these findings indicate the substrate specificity of *Drosophila* USP14 that may regulate protein stability.

### 3.3. Suppression of Late-Onset Retinal Degeneration by Drosophila USP14 in ninaE^G69D/+^ Flies

**Figure 3:**
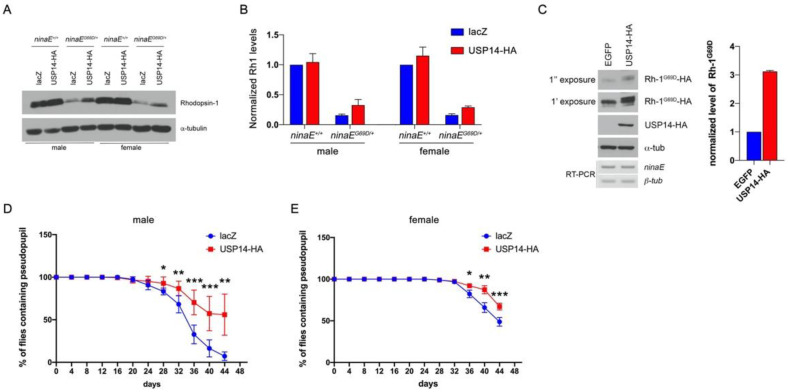
*Drosophila* USP14 suppresses late-onset retinal degeneration in *ninaE^G69D/+^* flies. (**A**) The level of Rhodopsin-1 in *Drosophila* adult head extracts with the indicated genotypes was determined by anti-Rh-1 and anti-α-tubulin (loading control). (**B**) Comparison of the normalized Rh-1 levels as shown in (**A**) (average of *n =* 4) with the value from the wild-type (lacZ in *ninaE^+/+^*) set at one. (**C**) The level of Rh-1^G69D^ was increased in USP14-overexpressing S2 cells. Rhodopsin levels were detected using the anti-HA antibody. (**D**,**E**) The evaluation of retinal degeneration was performed by the pseudopupil assay. For each genotype, the graph indicates the percentage of flies with intact pseudopupil determined by GFP pattern (*n* = 8). Specifically, lacZ or USP14 was overexpressed by the Rh1-Gal4 driver in the *ninaE* mutant. Overexpression of USP14 delayed the retinal degeneration of *ninaE^G69D/+^* flies. Error bars indicate ± SEM. *p*-values were obtained using Student’s *t*-test. * *p* < 0.05, ** *p* < 0.01, and *** *p* < 0.001.

To examine the effects of USP14 on misfolded protein degradation with a relevant disease model associated with ER stress, we used the *Drosophila* model of ADRP in which a mutant allele of the Rh-1 gene *ninaE^G69D^* has caused the age-related retinal degeneration phenotype associated with ER stress [24]. The level of Rh-1 protein is significantly reduced in the flies bearing *ninaE^G69D^*, which has a functional significance in retinal degeneration [25]. Similar to previous reports, the average Rh-1 level in heterozygotic flies bearing the *ninaE^G69D^* allele was reduced to approximately 20% of that in wild-type flies (Figure 3A,B; comparison of lanes 1 and 3). To determine whether USP14 contributes to this reduction in Rh-1 levels, it was overexpressed in the retina of wild-type or *ninaE^G69D/+^* flies using the Rh-1 driver. Overexpression of *Drosophila* USP14 in the *Drosophila* ADRP model flies restored the Rh-1 levels to approximately 34% of the expression in male wild-type flies (Figure 3A,B; lanes 3 and 4). We observed a similar result in female flies (Figure 3A,B; lanes 5‒8). Consistently, we observed that the level of mutant Rh-1 (Rh-1^G69D^) was increased in USP14-overexpressing S2 cells without affecting the mRNA level (Figure 3C). This result indicated that USP14 regulated the stability of Rh-1 in the ADRP model.

To further validate the role of USP14 in age-dependent disease progression, pseudopupil assay was performed. This assay is widely used to examine photoreceptor cell degeneration. The transgenic expression of GFP in the photoreceptors allows us to visualize the pseudopupil under fluorescent light. In male *ninaE^G69D/+^* flies expressing a control protein lacZ, the pseudopupil progressively disappeared beginning on day 20, with only 7% of flies demonstrating an intact pseudopupil on day 44 after eclosion (Figure 3D, blue line). Significantly, the expression of USP14 in the *ninaE^G69D/+^* background resulted in a delayed course of retinal degeneration with approximately 56% of the examined flies (*p* < 0.01) exhibiting pseudopupil on day 44 (Figure 3C, red line). A similar effect of USP14 on retinal degeneration was evident in female flies (Figure 3E), although the rate of retinal degeneration in females was delayed compared to that in males. In females, nearly 49% of the *ninaE^G69D/+^* flies expressing lacZ lost the deep pseudopupil by day 44. The USP14-expressing *ninaE^G69D/+^* flies demonstrated a delayed course of retinal degeneration with approximately 67% of flies exhibiting intact Rh1>GFP patterns (67.1 ± 4.079%, *p* < 0.001). We favor the interpretation that USP14 enhances the stability of both mutant Rh-1 and wild-type Rh-1, which helps traffic these proteins to rhabdomeres that harbor light-sensing organelles, which are equivalent to the outer segments of mammalian rod cells [26].

### 3.4. Dynamic Alteration of the Proteasome Complex in Response to ER Stress

**Figure 4:**
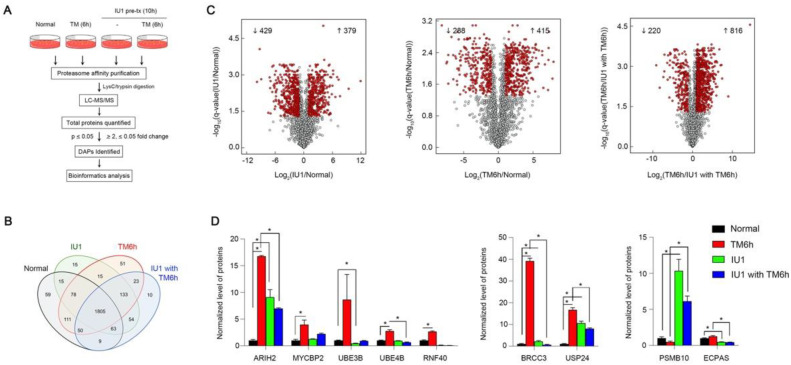
Proteasome dynamics in response to ER stress (**A**) an overview of the LC-MS/MS approach, which was used for the quantitation of the proteasome-interacting proteins under ER stress. (**B**) Venn diagram of the differentially associated proteins (DAPs) copurified with the proteasome. (**C**) Volcano plot (x-axis indicates the log_2_ of the fold changes, the y-axis indicates the negative decade logarithm of the significant value) for the three different comparisons. Proteins with a Benjamini-Hochberg FDR <0.05 and a log_2_ fold change ± 1 were considered differentially regulated (red dot). (**D**) Representative quantitation data comparing ubiquitination-related proteins and proteasome subunits in each condition. * *p* < 0.05.

We turned our attention to the function of USP14 in the proteasomal dynamics during ER stress. The proteasome is a diverse, dynamic, and heterogeneous supramolecular complex. The proteomes and the function of the proteasome can dynamically change depending on cell or tissue type, subcellular localization, extracellular stimuli, and pathological context [27,28,29,30]. As proteasomes are highly conserved [29], to examine the proteasomal dynamics under conditions of ER stress, human proteasomes were affinity-purified from HEK293 cells expressing biotin-tagged human 4 subunits. We analyzed the composition of the proteasome subunits and associated proteins in cells with or without TM-induced ER stress using mass spectrometry-based label-free quantification (Figure 4A). In addition, we tested the effect of IU1, a potent inhibitor of USP14, on proteasomal reorganization in cells subjected to ER stress. In total, 2491 proteins (2190 in normal cells, 2266 in 293 cells treated with TM for 6 h, 2178 in 293 cells treated with IU1 for 10 h, and 2147 in 293 cells subjected to pre-treatment with IU1 for 10 h followed by 6 h of TM treatment) were identified (Supplementary Table S1). However, 135 proteins in total were identified at least once from each experimental condition (Figure 4B). According to our criteria (ratio ≥ 2, ≤0.5, and Benjamini‒Hochberg FDR ≤ 0.05), differentially associated proteins were analyzed between the two samples described in Figure 4C and Supplementary Table S1. A total of 703 proteins demonstrated a significant change in interactions with the proteasome following 6 h of TM treatment compared to before the treatment. Among them, we observed that the interactions of some E3-ubiquitin ligases and proteasome adaptors with the proteasome changed dynamically in response to ER stress (Figure 4C,D and Supplementary Table S1). Specifically, ubiquitinating enzymes such as ARIH2, MYCBP2, UBE3B, UBE4B, and RFN40, accumulated in the proteasome in response to ER stress (Figure 4D). In addition, deubiquitinases such as BRCC3 and USP24 also accumulated in the proteasome during the 6-h TM treatment (Figure 4D). In contrast, IU1 altered the interaction of proteins with the proteasome under conditions of ER stress (Figure 4C). While PSMB10 accumulated in the proteasome upon treatment with IU1, the amount of ECPAS associated with proteasome decreased significantly (Figure 4D). In addition, Gene Ontology (GO) analysis indicates that mitochondria and spliceosome related proteins dynamically interacted with the proteasome and the association of some ERAD components with proteasome changed under ER stress (Supplementary Table S2). Although the exact mechanism of association of these proteins with the proteasome under conditions of ER stress remains to be explored, we believe that the structure of the proteasome complex is dynamically altered in response to ER stress. This change helps the cells cope with ER stress by modulating the degradation of misfolded proteins via interaction with multiple proteins associated with protein degradation.

## 4. Discussion

Here, we report the biological function of USP14 in *Drosophila*, which includes the regulation of ER stress, degradation of ERAD substrates, and disease progression in the *Drosophila* ADRP model. To investigate the role of USP14 in ER stress, we evaluated the activation of the UPR regulators, hsc3 and ATF4, which are the downstream effectors of the IRE1 and PERK pathways, respectively. As shown in Figure 1, the overexpression of USP14 in *Drosophila* S2 cells reduced the induction of the UPR regulators in the presence of TM- or thapsigargin-induced ER stress. To understand the mechanism underlying the USP14-mediated regulation of ER stress levels, we measured the proteasomal activity during ER stress, as UPS is directly associated with ER stress via ERAD [31,32]. Unexpectedly, the proteasome activity—evaluated based on the fluorogenic proteasome substrate Suc-LLVY-AMC—was decreased in *Drosophila* S2 cells exposed to ER stress (Supplementary Figure S2). Previous studies on mammalian cells and plants have reported enhanced proteasome activity during ER stress [33,34]. In contrast, a recent report indicates that the increased flux of misfolded protein burdens the proteasome via the ERAD pathway [35]. We speculate that the difference in our results in comparison with those of other groups can be attributed to the context, such as cell type or species. Since USP14 regulates both UPS and autophagy by regulating UVRAG degradation [36,37], we assumed that USP14 overexpression in *Drosophila* S2 cells experiencing ER stress does not regulate the proteasome activity but instead may activate other protective pathways such as autophagy to cope with ER stress. We observed the autophagic activity under conditions of ER stress using the anti-Atg8 antibody. The USP14 overexpressing S2 cells exhibited considerable Atg8a-II expression in response to ER stress (Supplementary Figure S5).

USP14 is known to contribute to the stability of cellular proteins by regulating proteasomal activity or its own activity. The allosteric regulation of the proteasome by USP14 is mediated by direct interactions with the ATPase ring in the proteasome, which interferes with the RPN11 function and inhibits the conformational change in the proteasome for proper substrate translocation [38]. In addition, USP14 is one of three proteasome-associated DUBs and promotes Ub recycling [39,40,41]. We tested the function of USP14 in the degradation of substrate proteins by examining NHK and ATZ expression. Overexpression of USP14 in S2 cells increased the expression of NHK but not that of ATZ. Conversely, USP14 knockdown resulted in reduced NHK expression (Figure 2). Given that USP14 interacts with NHK but not with ATZ, we assume that USP14 specifically recognizes its own substrate NHK, not ATZ, and thus stabilizes NHK through its enzymatic activity. Although these proteins are variants of A1AT, their fates and effects on cellular stress are different [10,23,42]. Thus, we hypothesize that the differential effect of USP14 on the ubiquitination of NHK and ATZ could be attributed to the structural features of the protein.

We found that NHK stabilized by USP14 may cause ER stress in S2 cells, which contradicts our conclusion that USP14 protects cells from ER stress. Mild ER stress can exert a positive effect on cells by inducing the expression of chaperones, making them resistant to subsequent challenge [43,44]. Considering that the activation of the ER stress marker xbp1-EGFP by NHK misexpression in *Drosophila* tissues was very weak, it is possible that NHK can lead to mild ER stress (data not shown). Thus, NHK expression in physiological or pathological contexts may lead cells to adapt to these perturbations by inducing the expression of protective genes. Therefore, USP14-stabilized NHK may increase cell survival.

In this study, USP14 also regulated the stability of Rh-1 and suppressed the retinal degeneration in a *Drosophila* ADRP model. Previous studies have demonstrated that mutant Rh-1 proteins interfere with the proper folding of wild-type Rh-1, resulting in enhanced proteasome-mediated degradation [13,45,46,47]. Conversely, the degradation of mutant Rh-1 by Hrd1 restores the trafficking of Rh-1 into rhabdomeres in ADRP [25]. Collectively, these findings support the hypothesis that *Drosophila* USP14 can increase the stability of both wild-type and mutant Rh-1 via its deubiquitinase activity, thereby increasing the overall levels of Rh-1 protein that are trafficked to the rhabdomeres. Finally, USP14 contributes to the suppression of retinal degeneration in a *Drosophila* model of ADRP.

We analyzed the assembly of proteasomes under ER stress. The interaction of the proteasome complex with some E3 ubiquitin ligases, deubiquitinases, and proteasome subunits dynamically changed under either ER stress or IU1-treated conditions (Figure 4D). Although the recognition of the proteasome substrate is mainly achieved by three ubiquitin receptors, namely Rpn1, Rpn10, and Rpn13, many different proteins escort the substrates to the proteasome [48,49]. Thus, we speculate that any specific protein complex associated with the proteasome recognizes specific proteasome substrates under ER stress without significantly changing the proteasome activity. This warrants further exploration. Given that the ERAD pathway is divided into sub-pathways according to the mutation site of the ERAD substrate [50,51], we believe that there may be diverse proteasome degradation pathways depending on the composition of the proteasome-interacting proteins.

## 5. Conclusions

In summary, we analyzed the function of USP14 in *Drosophila*. *Drosophila* USP14 contributed to reduced ER stress levels in *Drosophila* S2 cells. USP14 exerted protective effects in age-related retinal degeneration by regulating the stability of the Rh-1 protein. Given that many neurodegenerative diseases including ADRP are associated with protein toxicity, we believe that the modulation of USP14 could be a potential therapeutic strategy for the treatment of such diseases.

## Supplementary Materials

The following are available online at https://www.mdpi.com/2079-7737/9/10/332/s1, Figure S1: Quantitation of the normalized Hsc3 or ATF4 band intensity, Figure S2: Overexpression of USP14 decreased the levels of thapsigargin-induced ER stress, Figure S3: Knockdown efficiency of usp14 in *Drosophila* S2 cells, Figure S4: Kinetic measurement of proteasome activity, Figure S5: USP14 overexpression resulted in increased levels of Atg8a-II, Figure S6: Densitometric analyses and whole images of representative membranes for all the Western blots. Table S1: The summary of proteomic analysis, Table S2: Gene ontology (GO) analysis, Table S3: The lists on antibodies used in this study.
