# Supplementary material for: The Function of Drosophila USP14 in Endoplasmic Reticulum Stress and Retinal Degeneration in a Model for Autosomal Dominant Retinitis Pigmentosa
Source: Biology (Basel). 2020 Oct 12;9(10):332. doi: 10.3390/biology9100332 (PMC7650538; doi:10.3390/biology9100332)
Supplement: Supplementary file 1 [file biology-09-00332-s001.zip › biology-937178-Supplementary-figures.pdf]

biology

MDPI

Article

The Function of *Drosophila* USP14 in Endoplasmic Reticulum Stress and Retinal Degeneration in a Model for Autosomal Dominant Retinitis Pigmentosa

Jung-Eun Park, Thị Xuân Thùy Trần, Nayoung Park, Jeonghun Yeom, Kyunggon Kim and Min-Ji Kang

Supplementary Materials

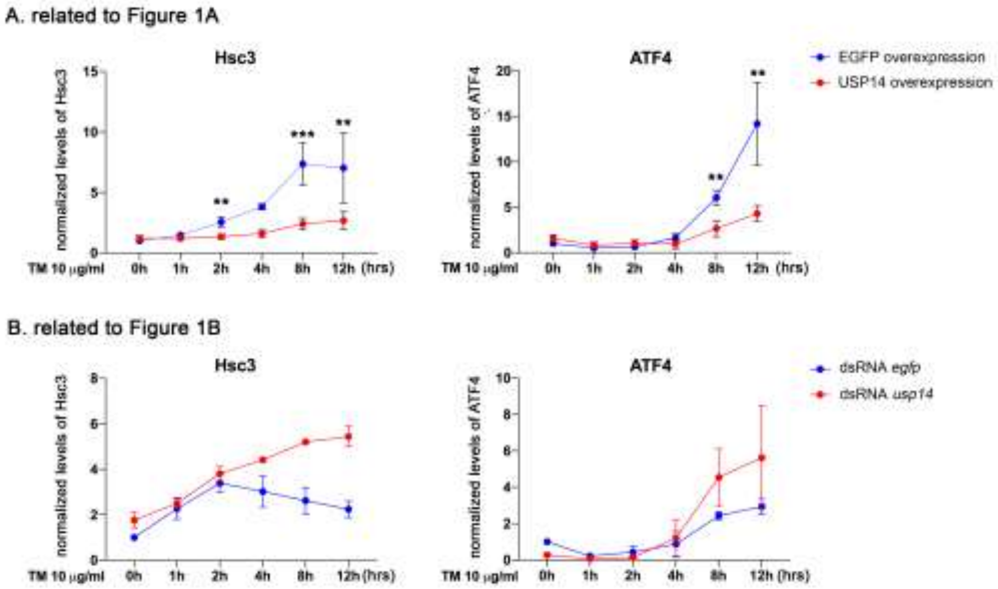

Figure S1. Quantification of the normalized Hsc3 or ATF4 band intensity, using ImageJ software. Error bars, ± SEM. (a) n > 3. The p values were calculated using Student's t test: ** $P < 0.05$, *** $P < 0.001$. (b) $n = 2$.

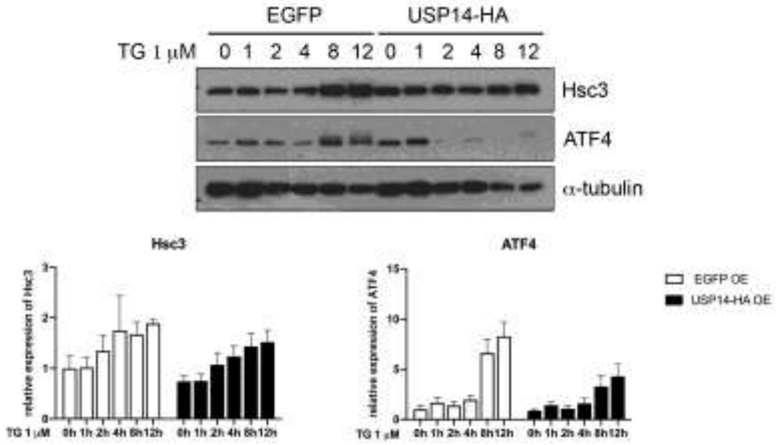

Figure S2. Overexpression of USP14 decreased the levels of thapsigargin-induced ER stress. (**a**) Control cells (EGFP overexpressing) and USP14 overexpressing S2 cells were treated with 1µM thapsigargin (TG) for the indicated intervals, and the extracts were analyzed for the expression of hsc3, ATF4, and α-tubulin using immunoblotting. (**b**) Quantification of the average normalized hsc3 or ATF4 band intensity using ImageJ. Error bars, ± SEM.

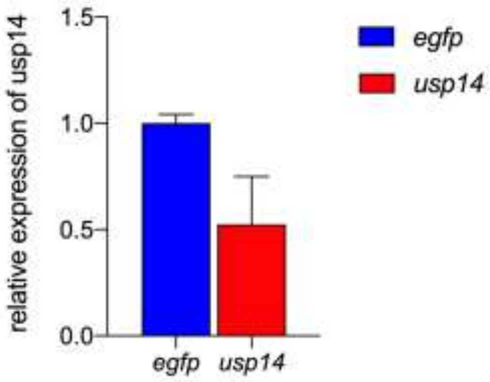

Figure S3. Knockdown efficiency of usp14 in Drosophila S2 cells As a control, RNAi against *egfp* was used. Error bars, ± SEM.

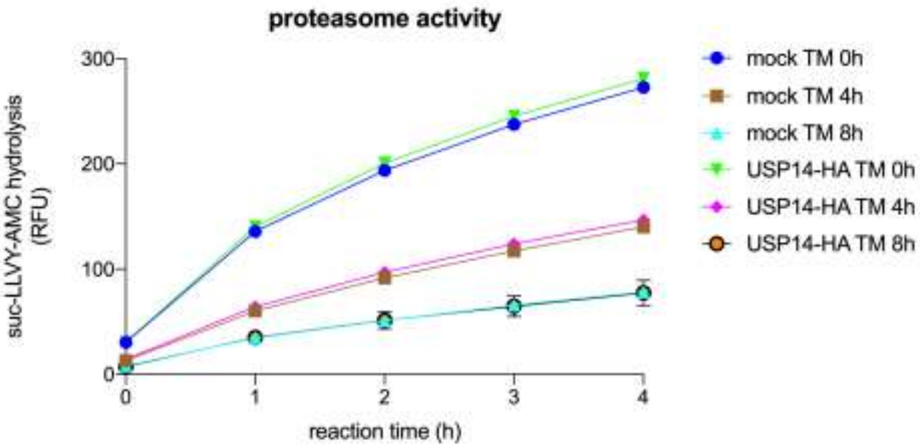

Figure S4. Kinetic measurement of proteasome activity. (**a** and **b**) *Drosophila* S2 cells expressing enhanced green fluorescent protein (EGFP, control) or USP14-HA were treated with 10 μg/ml tunicamycin (TM) for the indicated intervals. Proteasome activity was measured based on the hydrolysis of the Suc-LLVY-AMC fluorogenic substrate at the indicated time points in tunicamycin-treated *Drosophila* S2 cells. The values were normalized to basal activity, which was determined upon treatment with 20 μM MG132. RFU, relative fluorescence units.

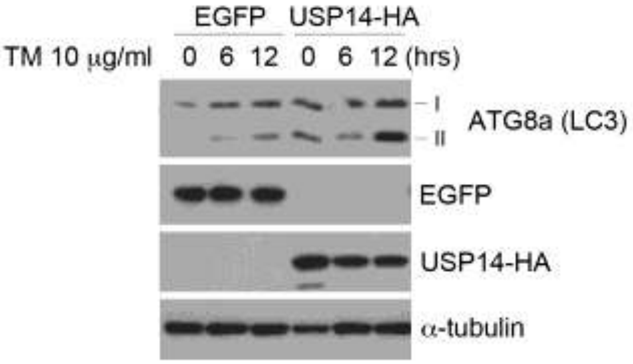

Figure S5. USP14 overexpression resulted in increased levels of Atg8a-II. *Drosophila* S2 cells were transfected with either EGFP (lanes 1–3) or USP14 (lanes 4–6) and treated with 10 μg/ml tunicamycin (TM) for the indicated intervals. The induction of autophagy was detected using the anti-Atg8 antibody. The increase in Atg8a-II levels was enhanced in USP14-overexpressing S2 cells.

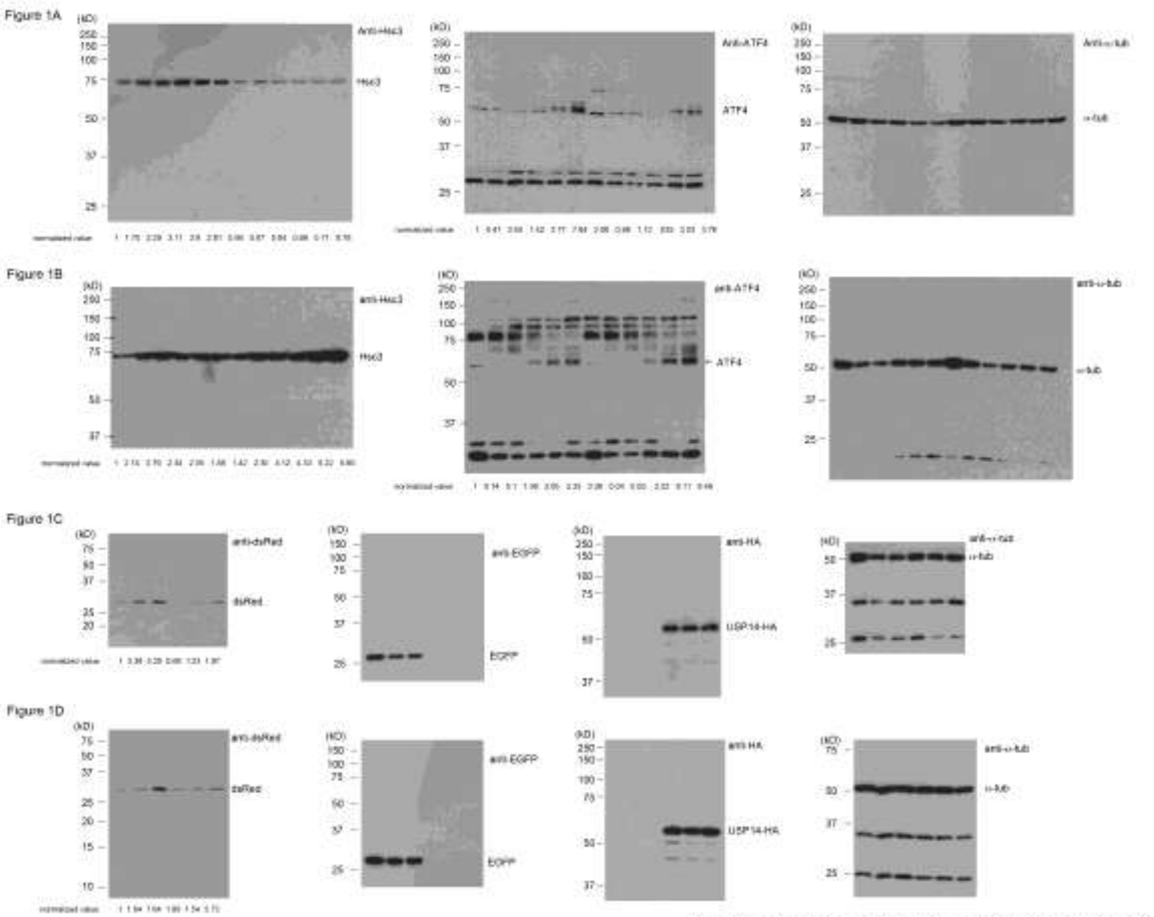

Supplementary Figure 6a, related to Figure 1

Figure 2A

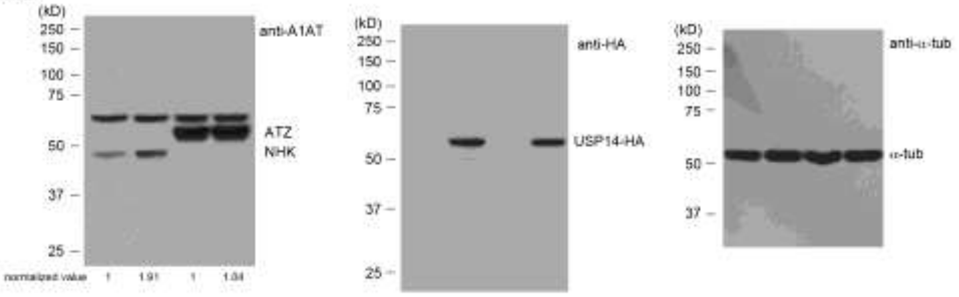

Figure 2B

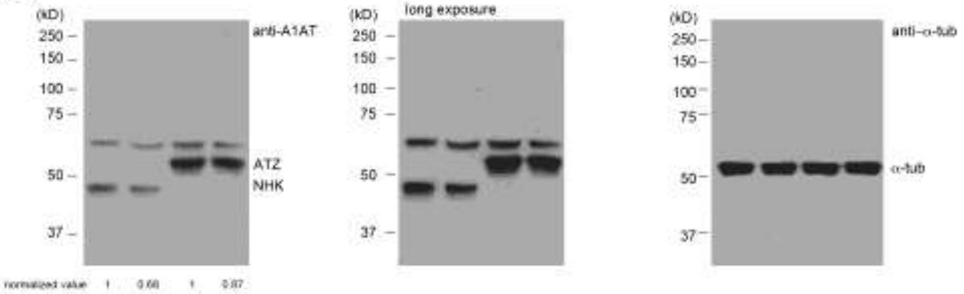

Figure 2C

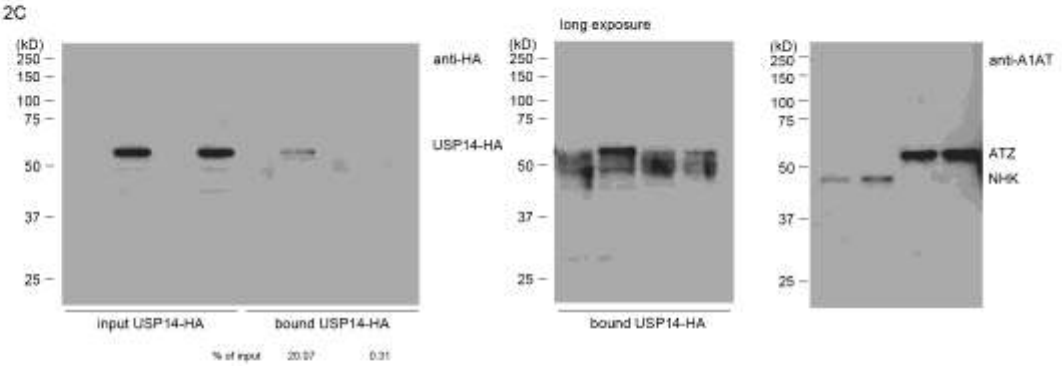

Supplementary Figure 6b, related to Figure 2

Figure 3A

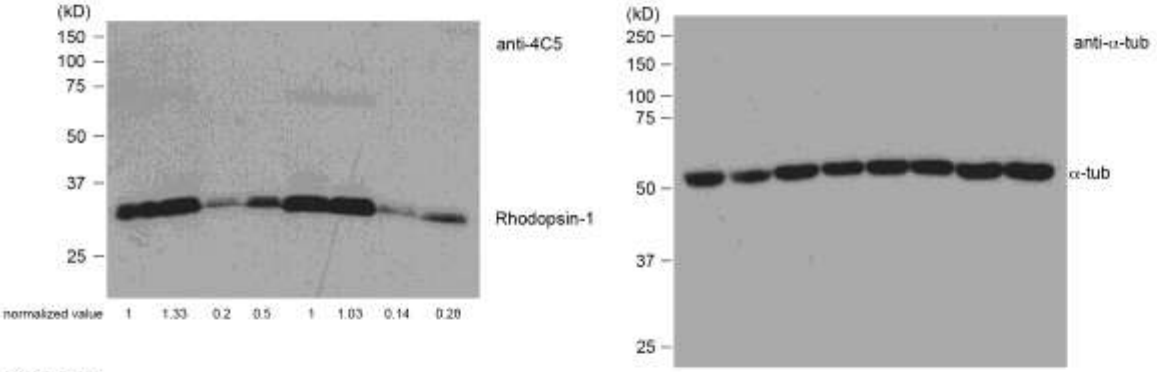

Figure 3C

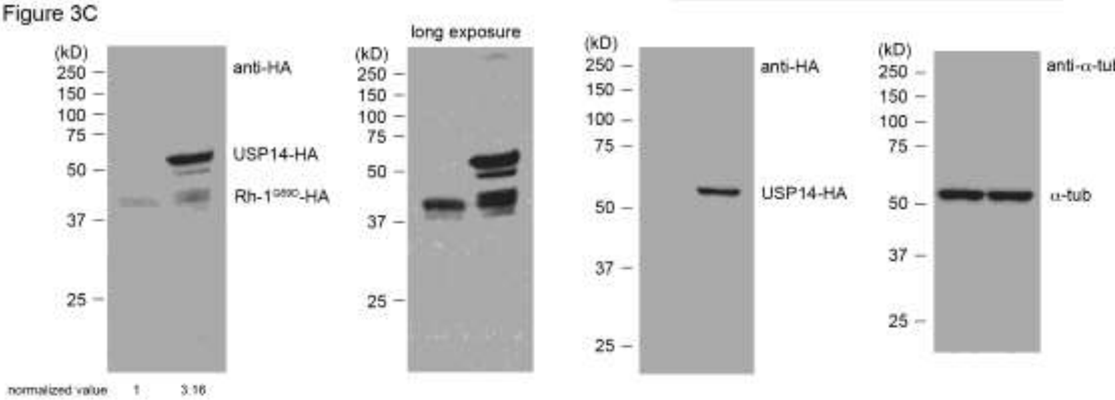

Supplementary Figure 6c, related to Figure 3

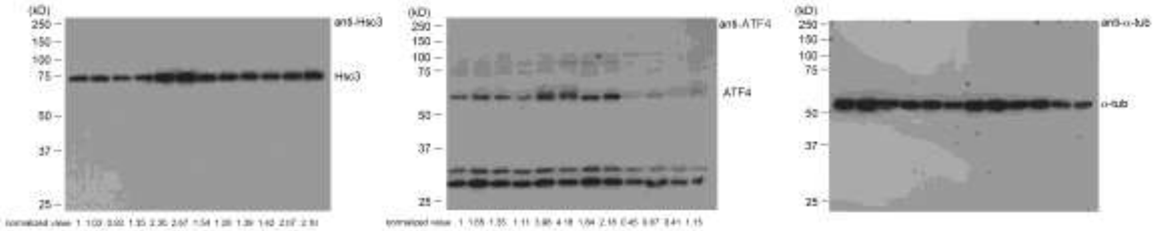

Supplementary Figure 6d, related to Figure S2

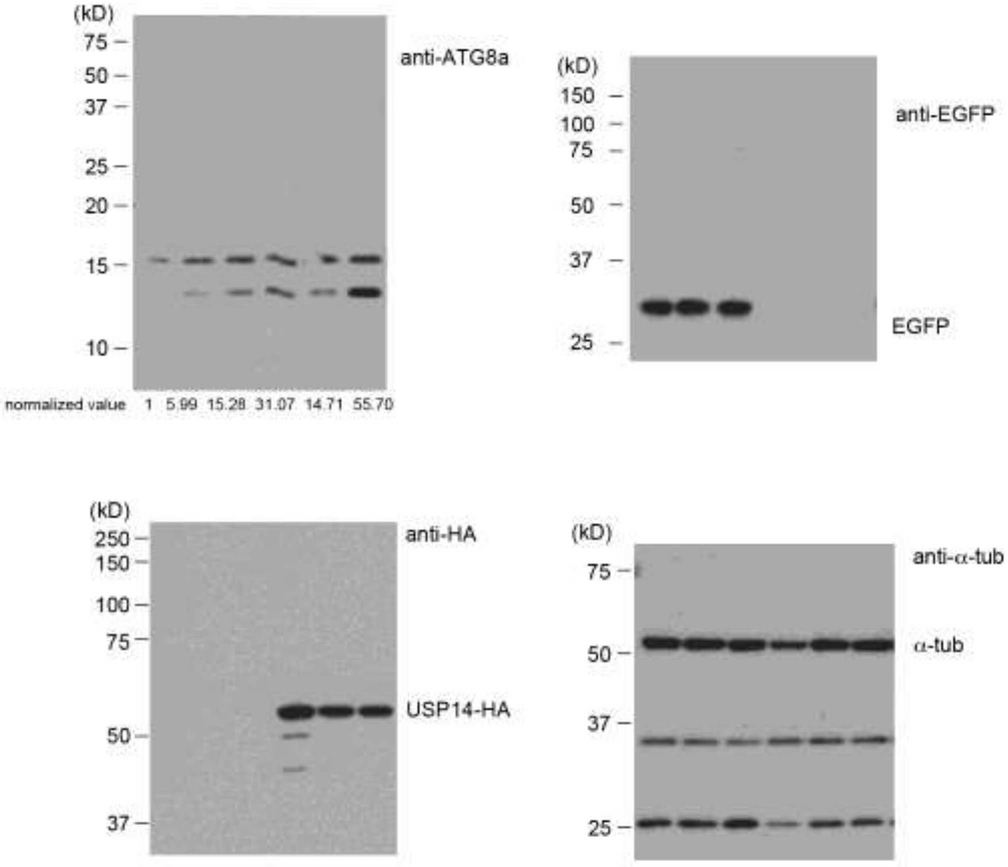

Supplementary Figure 6e, related to Figure S5

Figure S6. Densitometric analyses and whole images of representative membranes for all of the Western blots.
